# Continued spread of HIV among injecting drug users in southern Sichuan Province, China

**DOI:** 10.1186/1477-7517-4-6

**Published:** 2007-02-08

**Authors:** Lu Yin, Guangming Qin, Han-Zhu Qian, Yu Zhu, Wei Hu, Li Zhang, Kanglin Chen, Yunxia Wang, Shizhu Liu, Feng Zhou, Hui Xing, Yuhua Ruan, Ning Wang, Yiming Shao

**Affiliations:** 1 State Key Laboratory for Infectious Disease Prevention and Control, and National Center for AIDS/STD Control and Prevention, Chinese Center for Disease Control and Prevention, 27 Nanwei Road, Xuanwu District, Beijing 100050, China; 2 Sichuan Provincial Center for Disease Control and Prevention, 6 Zhongxue Road, Wuhou District, Chengdu, Sichuan Province 610031, China; 3 Department of Medicine/Division of Preventive Medicine, University of Alabama at Birmingham, Room 645, 1717 11th Avenue South Medical Towers Bld. Birmingham, Alabama 35205, USA; 4 Xichang Center for STD and Leprosy Control, 5 Chang'an Road, Zhangjiatunxiang, Xichang County, Sichuan Province 615000, China

## Abstract

**Objective:**

To estimate HIV prevalence among injecting drug users (IDUs) in a drug trafficking city in southwest Sichuan Province, China.

**Methods:**

A total of 314 IDUs was invited to participate in the cross-sectional survey in 2004 through community outreach recruitment and peer referrals. Blood sample was taken for HIV antibody testing and a structured questionnaire was administered to collect information on socio-demographics, drug using and sexual behaviors.

**Results:**

HIV prevalence among IDUs was 17.8% (56/314), about one half higher than that in previous survey in 2002 (11.3%, 43/379). Yi and other minority ethnicity (Odds ratio [OR], 3.1; 95% confidence interval [CI], 1.7–5.8; *P *< 0.001), and total times of sharing injecting equipments 1–9 times versus none, OR, 2.7; 95% CI 1.2–6.2; *P *= 0.02; and ≥10 times versus none, OR, 7.5; 95% CI, 3.2–17.7; *P *< 0.001) were independent risk factors for HIV infection.

**Conclusion:**

IDUs with high prevalence rates of HIV and equipment sharing behavior in the drug trafficking city may serve a source for further spread of HIV to other areas in China. The increasing trend of HIV epidemic among IDUs underscores the urgency of scaling up interventions.

## Background

Injecting drug use (IDU) has been the most important risk factor for HIV spread in China. The first HIV outbreak among IDUs was observed in 1989 in a bordering area in Southern Yunnan Province [1]; as of 2002, all 31 provinces, municipalities and autonomous regions in mainland China have reported HIV infection among IDUs [2]. A national epidemiological survey in 16 provinces in 2003 found that, on average, 54% drug users were IDUs and 7% IDUs were infected with HIV [Chinese Center for Disease Control, unpublished data]. It is estimated that about 44.3% of the 650,000 Chinese currently living with HIV/AIDS are drug users [2]. The relative importance of IDU in contribution to total reported HIV/AIDS cases has been declining over years as sexual transmission increases and HIV screening efforts among former plasma donors are enhanced; however, IDU is likely to remain as one major risk as drug use expands from rural bordering areas in Southwestern China to the whole country, especially to urban areas [3].

Xichang is a county-level city in Sichuan Province in Southern China and hosts a population of over 600,000 in total and 380,000 rural residents. The majority of the residents are Han ethnics and approximate 10% are Yi minority ethnics. Located on one major drug trafficking route [4], Xichang City has about 100,000 migrant people each year and nearly 2,500 registered local drug users. A community-based cross-sectional survey among 379 IDUs in 2002 found that 11.3% of IDUs in Xichang were infected with HIV [4], and a subsequent 12-month follow-up study showed a seroconversion rate of 3.17 per 100 person-years [5]. There are virtually no systemic population-based interventions, though some IDUs might receive research-oriented counseling services and even fewer participated in methadone maintenance therapy program which was started in Match 2004. We conducted this cross-sectional study in 2004 to estimate the trend of HIV epidemic among IDUs in this drug trafficking area.

## Methods

### Study design and study population

This cross-sectional study was conducted between May and July 2004 among IDUs in Xichang City, Sichuan Province. Known IDUs in the community were contacted directly by outreach workers and indirectly through words of mouth to invite IDUs to join the study. Snowball sampling was also employed as a recruitment strategy. A small financial incentive was given to participants who successfully referred peers to the study. Eligibility criteria required that participants be at least 18 years old and have injected drugs at least one time in the past 3 months, be willing to provide informed consent and give blood specimen for HIV testing, and have not participated in the cohort study conducted in 2002 [4,5], excluding those who were already known to have HIV infection before recruitment. The active injection of drugs was screened by eligibility questionnaire interview and verified by the presence of needle marks or medical record. Xichang Center for STD and Leprosy Control, whereby the study was conducted, has a database for all HIV-infected local residents in Xichang City, and a Participant Information File System (PIFS) software program was developed to record participants' name, locator information and study visits for this study as well as for previous cross-sectional and cohort studies [4,5], so that those who had been tested HIV-positive and/or had participated in the previous studies were identified during the eligibility screening and excluded for this study. After written informed consent was obtained, a structured questionnaire was administered by trained interviewers to collect information socio-demographics, drug use and sharing and sexual behaviors. A blood specimen was collected for testing of HIV antibody. All study participants were given HIV post-testing counseling, and those who were HIV positive were referred to China CARES project in Xichang City which provides comprehensive community-based HIV/AIDS services including voluntary testing and counseling, free antiretroviral therapy and treatment of opportunistic infections. Study protocol and informed consent were approved by the institutional review board of the National Center for AIDS/STD Control and Prevention of the China Center for Disease Control and Prevention.

### Laboratory analyses

Blood specimen was screened for HIV antibody by enzyme-linked immunosorbent assay (ELISA) (Beijing Wantai Biologic Medicine Co., China). Positive ELISA specimen was confirmed by an HIV-1/2 Western Blot immune assay (HIV Blot 2.2 WB; Genelabs Diagnositics, Singapore). Samples positive in both tests were considered HIV-positive.

### Statistical analysis

Questionnaire data were double-entered and compared with Epi Data software (Epi Data 3.1 for Windows; The Epi Data Association Odense, Denmark). Then, the data were converted and analyzed using Statistical Analysis System (SAS 9.1 for Windows; SAS Institute Inc., NC). Univariate analyses including Chi-square test, Fisher's exact test, or Mantel-Haenszel chi-square test were performed to evaluate the association of HIV infection with socio-demographic, drug using and sexual behaviors. Those variables significant in univariate analyses (*P *≤ 0.10) were included in a multivariate logistic regression model. The final model was constructed by eliminating non-significant variables in a stepwise manner, identifying variables that were independently associated with HIV seropositivity.

## Results

### Socio-demographic characteristics, drug using and sexual behaviors

A total of 316 IDUs were screened, one person did not meet eligibility criteria and one person refused to participate; therefore, 314 subjects were included in the analyses. 247 were referred by seeds or peers (78.7%), others by outreach workers. The average age was 29 (range 18–46) years; 85.7% were males; 38.8% were Yi or other minority ethnics. Education distribution was illiteracy (14.6%), primary school (23.6%) and junior high school (44.9%). About half (49.4%) were never married, and 23.6% were divorced. 61.2% of subjects were unemployed and 26.0% owned a house.

The average age of beginning to use drugs, injecting drugs and sharing injection equipments (including needles or syringes) were 22, 25 and 26 years, respectively. In the past 3 months, 64.3% used heroin as frequently as ≥7 times per week. Of all subjects, 62.7% reported a history of sharing equipments. Reasons for sharing equipments included: "do not know where to obtain", "inconvenient to obtain", and "high price of syringes and needles." Fifty-nine percent of subjects reported having heterosexual activities and none having same-sex contacts in the past six months, and 24.2% had new sex partners; 20.4% gave money for sex and 7.3% received money for sex. Among 67 subjects who had casual and/or commercial sexual partners in the last month, 10.5% reported using condoms consistently.

### HIV prevalence and risk factors

Of 314 participants, 56 (17.8%) were infected with HIV. In univariate analyses, factors significantly associated with HIV infection at a level of 0.10 are sex, marital status, ethnicity, total times of sharing injecting equipments in the past, total number of partners sharing injecting equipments in the past, having new partners sharing injecting equipments in last 3 months, sharing needles and syringes, rinse water, and cookers in the last 3 months, having heterosexual intercourses and not having a primary sexual partner in the last 6 months (Table 1, 2, 3).

**Table 1:** Socio-demographic Factors Associated with HIV seropositivity among IDUs in Xichang City, Sichuan Province, China

Factor	N	% HIV+ [N]	Odds Ratio [95% CI]	*P*-value
Total	314	17.8 [56]		
Referred by peers				
No	65	15.4 [10]	1.0	
Yes	249	18.5 [46]	1.25 [0.59, 2.63]	0.56
Sex				
Male	269	19.7 [53]	1.0	
Female	45	6.7 [3]	0.29 [0.09, 0.98]	0.03
Age				
<29 years	136	21.3 [29]	1.0	
≥29 years	178	15.2 [27]	0.66 [0.37, 1.18]	0.16
Ethnicity				
Han	192	11.5 [22]	1.0	
Other	122	27.9 [34]	2.98 [1.65, 5.41]	<0.01
Years of education				
≤6	120	21.7 [26]	1.0	
>6	194	15.5 [30]	0.66 [0.37, 1.18]	0.16
Employed				
Yes	192	19.3 [37]	1.0	
No	122	15.6 [19]	0.77 [0.42, 1.42]	0.40
Marital Status				
Married or cohabited	83	10.8 [9]	1.0	
other	231	20.4 [47]	2.10 [0.98, 4.50]	0.05
Owning a house				
No	230	17.0 [39]	1.0	
Yes	84	20.2 [17]	1.24 [0.66, 0.34]	0.50
Yearly income in 2003				
<1,000 dollars	119	16.0 [19]	1.0	
≥1,000 dollars	195	19.0 [37]	1.23 [0.67, 2.26]	0.49
MMT participation				
No	259	18.5 [48]	1.0	
Yes	55	14.6 [8]	0.75 [0.33, 1.69]	0.48

MMT, methadone maintenance therapy.

**Table 2:** Drug Use Behavioral Factors Associated with HIV Seropositivity among IDUs in Xichang City, Sichuan Province, China

Factor	N	% HIV+ [N]	Odds Ratio [95% CI]	*p*-value
During of drug injection				
<3 years	162	18.5 [30]	1.0	
≥3 years	152	17.1 [26]	0.91 [0.51, 1.62]	0.74
Using heroin in the last 3 months				
<7 times/week	112	17.9 [20]	1.0	
≥7 times/week	202	17.8 [36]	1.00 [0.54, 1.82]	0.99
Using heroin plus other drugs in the last 3 months				
<7 times/week	123	14.63 [18]	1.0	
≥7 times/week	191	19.9 [38]	1.45 [0.78, 2.68]	0.23
Injecting drugs in the last 3 months				
<7 times/week	35	11.4 [4]	1.0	
≥7 times/week	279	18.6 [52]	1.78 [0.60, 5.25]	0.29
Total times of sharing injecting equipments in the past				
0 times	117	7.7 [9]	1.0	
1–9 times	130	16.9 [22]	2.44 [1.08, 5.55]	
≥10 times	67	37.3 [25]	7.14 [3.08, 16.56]	<0.01
Number of partners sharing injecting equipments in the past				
0 persons	117	7.7 [9]	1.0	
1–9 persons	157	22.9 [36]	3.57 [1.64, 7.75]	
≥10 persons	40	27.5 [11]	4.55 [1.72, 12.03]	<0.01
Having new partners sharing injecting equipments in the last 3 months				
No	251	14.7 [37]	1.0	
Yes	63	30.2 [19]	2.50 [1.32, 4.74]	<0.01
Sharing needles and syringes in the last 3 months				
No	197	14.7 [29]	1.0	
Yes	117	23.1 [27]	1.74 [0.97, 3.11]	0.06
Sharing rinse water in the last 3 months				
No	233	15.4 [36]	1.0	
Yes	81	24.7 [20]	1.79 [0.97, 3.33]	0.06
Sharing cookers in the last 3 months				
No	234	15.4 [36]	1.0	
Yes	80	25.0 [20]	1.83 [0.99, 3.40]	0.05
Sharing cottons in the last 3 months				
No	301	17.3 [52]	1.0	
Yes	13	30.8 [4]	2.13 [0.63, 7.17]	0.24
Front-/back-loading in the last 3 months				
No	278	17.6 [49]	1.0	
Yes	36	19.4 [7]	1.13 [0.47, 2.72]	0.79

**Table 3:** Sexual Behavioral Factors Associated with HIV Seropositivity among IDUs in Xichang City, Sichuan Province, China

Factor	N	% HIV+ [N]	Odds Ratio [95% CI]	*p*-value
Having heterosexual intercourses in the last 6 months				
No	130	22.3 [29]	1.0	
Yes	184	14.7 [27]	0.60 [0.34, 1.07]	0.08
Having a primary sex partner in the last 6 months				
No	218	21.1 [46]	1.0	
Yes	96	10.4 [10]	0.43 [0.21, 0.90]	0.02
Having unprotected sex with a primary sex partner in last month				
No	252	19.0 [48]	1.0	
Yes	62	12.9 [8]	0.63 [0.28, 1.41]	0.26
Having non-primary sex partners in the last 6 months				
No	204	18.6 [38]	1.0	
Yes	110	16.4 [18]	0.85 [0.46, 1.58]	0.62
Having unprotected sex with non-primary sex partners in last month				
No	254	16.1 [41]	1.0	
Yes	60	25.0 [15]	1.73 [0.88, 3.40]	0.11
Giving money for sex in the last 6 months				
No	250	19.2 [48]	1.0	
Yes	64	12.5 [8]	0.60 [0.27, 1.34]	0.21
Receiving money for sex in the last 6 months				
No	291	18.2 [53]	1.0	
Yes	23	13.0 [3]	0.67 [0.19, 2.35]	0.78
Having new sex partners in the last 6 months				
No	238	18.5 [44]	1.0	
Yes	76	15.8 [12]	0.83 [0.41, 1.66]	0.59

All variables significant in univariate analyses were entered to multivariate logistic regression models. A final model was constructed with the following variables independently associated with HIV infection: Yi and other minority ethnicity (odds ratio [OR], 3.1; 95% confidence interval [CI], 1.7–5.8; *P *< 0.001) and total times of sharing injecting equipments in the past (1–9 times versus none, OR, 2.7; 95% CI 1.2–6.2; *P *= 0.02; and ≥10 times versus none, OR, 7.5; 95% CI, 3.2–17.7; *P *< 0.001) (Table 4).

**Table 4:** Multivariate Logistic Regression Analysis of Factors Associated with HIV Seropositivity

Factor	OR [95% CI]	*p*-value
Ethnicity (Minorities versus Han majority)	3.11 [1.67, 5.80]	<0.001
Total times of sharing injecting equipments in the last 3 months		
1–9 times vs. 0 times	2.70 [1.17, 6.22]	0.02
At least 10 times vs. 0 times	7.45 [3.15, 17.65]	<0.001

## Discussion

Given the same recruitment methods and participation eligibility criteria were employed, this study should be comparable with the first community-based survey in 2002 in estimating HIV prevalence among IDUs in the study site. HIV prevalence rate in 2004 (17.8%) increases by over 50% than that in 2002 (11.4%, *P *= 0.02), which is consistent with the finding of 3.17 per 100 person-years seroconversion rate among IDU cohort during years of 2002–2003 [5]. The high HIV prevalence rate and prevalent equipment sharing practices are worrisome. Studies have shown that once HIV prevalence among a high-risk IDU population reaches 20%, HIV epidemic can become self-perpetuating and a modest level of risk behaviors may lead to a substantial rate of infection [6,7]. Furthermore, Xichang is located in one major drug trafficking route, and HIV-infected IDUs in Xichang may become a source for further HIV spread to other geographic areas. China has initiated harm reduction projects including methadone maintenance therapy (MMT) and needle exchange programs. Xichang Center for STD and Leprosy Control is one of the first 8 clinics in the country to provide pilot MMT service since early 2004. Limited experiences show a high drop-out rate of MMT. As China plans to scale up harm reduction projects and expand MMT service to 1000 clinics across the country within five years [8], research is urgently needed to improve the efficacy of MMT itself as well as adding counseling and behavioral intervention components.

The frequency of sharing injecting equipments is an independent risk factor for HIV infection and more times of sharing is associated with higher risk of infection. Beside direct sharing of needles or syringes [9-13], indirect sharing of injecting equipments, including cottons, rinse water, cookers, and front/backloading were also reported as risk factor for HIV infection in other studies [14,15], but were not confirmed in our study. "Do not know where to obtain", "inconvenient to obtain" and "high price of syringes and needles" were reported as reasons of sharing equipments, suggesting that provision of clean equipments through needle exchange programs might reduce risk behaviors among IDUs. Another independent factor for HIV infection is Yi or other minority ethnicities. Majority of Yi and other minority people are living in remote rural areas of Xichang City and are less like to receive health education and services. Due to relatively poorer economic status and higher rate of unemployment, Yi ethnic people are more likely to be involved in drug smuggling and abuse. Intervention projects should give high priority to ethnic minority people.

This study has several limitations. First, subjects were not randomly selected; targeted sampling depends on chain-referrals (snowball sampling). IDUs who participated in the study may differ with those who did not; therefore, the estimate HIV prevalence may not exactly reflect the true rate in this study community. However, the sampling method is same as that in 2002 survey, and HIV prevalence rates should be comparable in these two cross-sectional surveys. Second, drug using and sexual behavioral data were based on self-reports, and recall bias and social desirability bias are possible. However, the primary outcome of this study – HIV prevalence – is based on laboratory testing. The continued increase of HIV epidemic among IDUs in Xichang City suggests that comprehensive interventions for reducing high-risk drug taking and equipment sharing behaviors are urgently needed.

## Competing interests

The author(s) declare that they have no competing interests.

## Authors' contributions

Lu Yin conducted data collection and analyses and drafted the manuscript. Guangming Qin was involved in study design and laboratory test. Li Zhang, Shizhu Liu, Feng Zhou, Kanglin Chen, Yunxia Wang assisted in designing the study and collecting data. Yu Zhu and Wei Hu assisted in data collection. Han-Zhu Qian and Yuhua Ruan designed the study and provided guidance on data analyses and manuscript revision. Hui Xing, Ning Wang and Yiming Shao were study consultants. Yuhua Ruan and Yiming Shao were principle investigators (PI) of these studies. All authors read and approved the final manuscript.
